# Relationship between influenza-related experience and current vaccination outcome

**DOI:** 10.1186/s12889-024-21263-5

**Published:** 2025-01-16

**Authors:** Yunwei Gai

**Affiliations:** Economics Division, Babson College, 231 Forest Street, Wellesley, MA United States

**Keywords:** Influenza vaccination, Influenza infection, Health care disparity, Medical expenditure panel survey

## Abstract

**Background:**

This study investigated how a person’s influenza-related experience, together with demographic, socioeconomic, and health-related factors, was associated with their current vaccination decisions.

**Methods:**

The analysis used ten panels of the Medical Expenditure Panel Survey (MEPS) from 2006 to 2016. Linear and logistic probability models were estimated to predict influenza vaccination using a person’s vaccination status in the previous year and history of influenza infection, adjusting for demographics, socioeconomic variables, general health status, and healthcare access. The models used two-way and three-way interactions with race/ethnicity, income, education, health status, and elderly status to examine changing relationships of flu-related experience across these variables.

**Results:**

Previous vaccination was the most important predictor, with an increase of 63.0–71.8% probability of vaccination in the next year. Infection history could either increase or decrease the impact of past vaccination depending on race/ethnicity, income, education level, health status, and age. There were significant disparities across demographic, socioeconomic, and health-related variables.

**Conclusion:**

Vaccination promotion efforts could focus on those who have not been vaccinated in the past and on specific sub-populations, such as people who are Hispanic, people with lower education levels, the population aged 65 and above, and families with low-income levels. Although past infection is a predictor for some population groups, its magnitude is small and is often not a significant determinant.

**Supplementary Information:**

The online version contains supplementary material available at 10.1186/s12889-024-21263-5.

## Background

Millions of people are sickened by influenza every year. The Centers for Disease Control and Prevention (CDC) estimate that influenza has resulted in 9 million–45 million illnesses, 140,000–810,000 hospitalizations, and 12,000–61,000 deaths annually since 2010 [1]. Recognizing the cost-effectiveness of influenza vaccination [2], the 2010 Patient Protection and Affordable Care Act required insurance policies to cover it without copayment or co-insurance.

Despite these efforts, vaccination rates remain low. For example, *Healthy People 2030* calls for achieving vaccination rates of 70% among persons six months and older. However, the actual rate during the 2020–2021 influenza season was 52.1% [3], with significant racial/ethnic and income disparities [4].

Many studies have explored predictors for vaccination. This area of research is critical for understanding different vaccination rates and designing sound public health policies to promote flu vaccination and other preventive care. These predictors often include individual factors such as individual socioeconomic and demographic variables; neighborhood and contextual factors such as racial and ethnic composition and local healthcare resources and policies; and macroeconomic conditions such as local market conditions and unemployment rates. In addition, researchers and practitioners point out that some significant barriers include the lack of vaccination knowledge, resistance attitudes, and disbelief [5–10].

A qualitative meta-analysis of 29 CDC-sponsored studies found personal experience can be both a facilitator and a barrier [5]. It depends on their experience’s severity, vaccination status, demographics, and socioeconomic factors. For instance, people who have experienced severe influenza cases are more motivated to get vaccinated to prevent future infections, and this observation is particularly apparent among the population group over 50 years of age. The younger and healthier population group may view experience with flu, especially a minor case, as proof of their immunity and a reason for not getting vaccinated.

Besides being a facilitator and a barrier by itself, previous experience with flu can affect other facilitators and obstacles such as “perceived susceptibility or health threat,” “flu vaccine recommendations do not apply to me,” “not susceptible to serious illness” and “influenza is a ‘manageable’ illness” [5]. The authors further concluded that because knowledge, attitudes, and beliefs (KABs) are often rooted in personal experience rather than population-level statistics, promotion materials and messages that resonated with their experiences could be more effective than other forms of communication. Other studies, primarily based on interviews, had similar conclusions [11–15].

Empirical studies use various data types and methods to establish the highly significant relationship between previous and current year’s vaccinations [11–14, 16, 17]. Several review papers summarized findings from these empirical studies. In a 2016 paper [17], the authors conducted a systematic literature review of 23 quantitative studies selected from EMBASE, MEDLINE, Cochrane Library, and Electronic Theses Online Service. These selected studies represented data collection from 1997 to 2012 in 18 countries. They covered different data types and methodologies, including 18 cross-sectional surveys, one case-control study, one randomized control trial, two longitudinal studies, and one meta-analysis. One of the main conclusions from this review paper is that previous influenza vaccination was a good predictor for subsequent vaccination. In a 2018 paper [13], the authors reviewed empirical studies from 1985 to 2016. Studies under review collected data in various ways, such as in-depth interviews, focus groups, and surveys by Internet, telephone or mail. The review identified past vaccination history as one of the most important determinants. A systematic review of 470 articles from 2005 to 2016 reached the same conclusion on the importance of past vaccination [17].

Previous work has sample sizes ranging from tens to a few thousand individuals. The data were often collected in small areas such as workplaces, primary care sites, clinics, towns, and cities. They may not simultaneously control other confounding factors such as family income, education level, general health status, and insurance coverage. Therefore, it is challenging to generalize previous findings at the national level. In contrast to much of the earlier work, this study used 1) a nationally representative sample over ten years to explore how a person’s past influenza-related experience may interact with other factors to influence their current decisions on vaccination. 2) controlled for individual demographics, socioeconomic factors, and health-related information. 3) used validated records of influenza diagnoses and vaccination status by healthcare providers rather than purely interview responses.

Another contribution of this study is examining the interactions between influenza-related experience and individual factors such as age, education, income, and race and ethnicity. Studies have shown that people’s risk attitude towards influenza and their concerns about vaccine side effects vary significantly across these factors [5, 9].

## Methods

### Data

Data in this study came from the ten panels (panel 11 to panel 20 from 2006 to 2016) of the Medical Expenditure Panel Survey (MEPS). The U.S. Public Health Service conducts MEPS through the Agency for Healthcare Research and Quality (AHRQ) and CDC. On average, there are close to 20,000 observations in each panel. Observations were restricted to adults aged 18 and above because only this population group was asked the flu vaccination question.

Each panel interviewed the same household and noninstitutionalized individuals five times over two-and-a-half years. For example, the first round of interviews in the 2010 panel was conducted in January 2010. Individuals were interviewed the second time between February and August 2010. The third round of interviews was carried out between August and December 2010. The fourth round was conducted between January and June 2011. The final round was between July and December 2011. A new group of participants was recruited for the 2011 panel in January 2011 and was interviewed five times from 2011 to 2012. The time frame changes slightly from panel to panel, but the main structure remains similar. Detailed panel design and data collection process are available at https://meps.ahrq.gov/mepsweb/survey_comp/hc_data_collection.jsp.

This study used the household full-year longitudinal file and the medical conditions file. Both are publicly available and can be downloaded directly from MEPS^1^. The downloaded files are in SAS or ASCII formats. The websites also provide SAS, STATA, and SPSS programming statements for importing and processing datasets. In this study, the individuals’ socioeconomic variables, demographics, general health, insurance, healthcare access, and vaccination status are from the household file. Their influenza infection history and medical conditions came from the medical conditions file.

### Variables

MEPS websites have detailed documentation and codebooks for each dataset. For example, the codebook for Panel 18 conducted during the 2013–2014 period is available at https://meps.ahrq.gov/mepsweb/data_stats/download_data_files_codebook.jsp?PUFId=H172. The current and past vaccination status is coded as FLUSHT3 and FLUSHT5, respectively. They are measured by survey questions on whether a person is vaccinated in the first year and the second year. In this study, the binary indicators for a person’s vaccination status are determined by their answers to the two questions. The other variables are selected similarly.

Influenza infection history came from the medical conditions file in MEPS. Each record in this file represents a medical condition reported by the interviewee. MEPS uses Clinical Classification Software (CCS) to identify influenza cases. CCS is a tool developed by the AHRQ for clustering diagnoses into a manageable number of clinically meaningful policy-relevant categories. The CCS for influenza is 123, which covers the International Classification of Diseases, 9th revision, or ICD-9 codes 4870, 4871, 4878, 488, 4880, 48801, 48802, 48809, 4881, 48811, 48812, 48819, 48881, 48882, and 48889.

Demographic, socioeconomic variables, general health status, and healthcare access came from the household file. Demographic variables include age, gender, race and ethnicity, geographic regions, family size, and marital status. Age is divided into two categories: 18–64 (reference group in the model) and age 65 and above. Race and ethnicity cover four categories: non-Hispanic white respondents (reference group in the model), non-Hispanic black respondents, Hispanic respondents, and respondents of non-Hispanic other races. Four geographic regions include the Northeast (reference group), Midwest, South, and West.

Socioeconomic variables include three levels of family income (low income as the reference group, middle income, and high income), employment status, and two education levels (less than college as the reference group, and college or more). The general health status is measured by a person’s overweight or obesity status, three levels of self-reported overall health (fair or poor as the reference, good, and very good or excellent), and whether their physicians reported a priority medical condition.

Priority conditions, as specified by AHRQ, include hypertension, heart disease, high cholesterol, emphysema, chronic bronchitis, diabetes, cancer, arthritis, asthma, attention-deficit/hyperactivity disorder, and stroke. AHRQ categorizes them as priority conditions because of their prevalence, expense, relevance to policy, and because standards for care for them have been developed [18, 19].

A person’s lack of access to healthcare is measured by a dichotomous variable. It equals one if a respondent answered yes to any of the following questions: (1) Did you lack a usual source of care if you were sick or needed advice about your health? (2) Were you unable to or delayed getting necessary medical care? (3) Were you unable to or delayed getting necessary dental care? and (4) Were you unable to or delayed getting the necessary prescription medicine?

This study defined seven insurance patterns to measure a person’s insurance status because insurance instability, different uninsured spells, and interruption patterns can affect health outcomes differently [20, 21]. MEPS records respondents’ monthly public and private coverage. Private coverage includes employer-provided health insurance (EPHI), self-purchased insurance (such as from the health insurance exchange), and other types of private insurance. Public insurance includes Medicare, Medicaid/SCHIP, TRICARE/CHAMPVA, and other public sources. Based on these records, the seven patterns are (1) always insured, (2) single gap, (3) transition into coverage, (4) transition out of coverage, (5) temporary coverage, (6) repeatedly uninsured, and (7) always uninsured (the reference category).

Finally, all models included the year and quarterly indicators to capture the flu season’s severity and other seasonal variations.

### Statistical analysis

Two sets of linear probability models were estimated to analyze the relationship between influenza-related experiences and vaccination outcomes. All models include previously mentioned demographic, socioeconomic variables, general health status, and healthcare access as covariates.

The first model, shown in Eq. 1, includes a person’s previous vaccination, infection, and their interaction term. The coefficient for the interaction term examines whether the impact of past vaccination can be affected by their previous influenza infection.

1 $$Vaccinated_{i,t}\;=\beta_0\;+\;\beta_1X_{i,1}\;+\;\beta_2X_{i,2}\;+\;\beta_3PastVac_{i,t-1}\;+\;\beta_4PastFlu_{i,t-1}\;+\;\beta_5PastVac_{i,t-1}\;\times PastFlu_{i,t-1}\;+\;\varepsilon_i$$

Where *Vaccinated*_*i,t*_ is a dummy variable that equals one if individual *i* has been vaccinated at time *t*. *X*_*i,1*_ is the set of respondents’ individual and family characteristics; *X*_*i,2*_ is the set of the year and quarterly indicators to capture the flu season’s severity.

*PastVac*_*i,t-1*_ indicates individual *i* vaccination status in year *t-1*. It takes the value of one if individual *i* was vaccinated in year *t-1*. *PastFlu*_*i,t-1*_ indicates individual *i* influenza status in year *t-1*. It takes the value of one if the individual *i* had influenza in year *t-1*. The coefficient, *β*_*5*_ for the interaction term, *PastVac*_*i,t-1*_*×PastFlu*_*i,t-1*_ captures whether past influenza infection affects the impact of past vaccination on current vaccination status.

The second set of models, shown in Eq. 2, follows similar specifications and adds a three-way interaction term. The three-way interaction term is among a person’s previous vaccination, infection, and one of the following five factors: race/ethnicity, income, education, health, and elderly status. Depending on which factor is used, five equations will be estimated.

2 $$Vaccinated_{i,t}\;=\beta_0\;+\;\beta_1X_{i,1}\;+\;\beta_2X_{i,2}\;+\;\beta_3PastVac_{i,t-1}\;+\;\beta_4PastFlu_{i,t-1}\;+\;\beta_5Z_i\;+\;\beta_6PastVac_{i,t-\mathit1}\;\times PastFlu_{i,t-\mathit1}\;+\;\beta_7PastVac_{i,t-\mathit1}\times Z_i\mathit\;+\mathit\;\beta_{\mathit8}PastFlu_{\mathit i\mathit,\mathit t\mathit-\mathit1}\mathit\times Z_{\mathit i}\mathit\;+\;\beta_{\mathit9}PastVac_{\mathit i\mathit,\mathit t\mathit-\mathit1}\mathit\;\times PastFlu_{\mathit i\mathit,\mathit t\mathit-\mathit1}\mathit\times Z_{\mathit i\mathit\;}\mathit\;+\varepsilon_{\mathit i}\mathit\;\;$$

Where *Z*_*i*_ is one of the five factors. For example, this study has four race/ethnicity categories: non-Hispanic white respondents (reference group), Hispanic respondents, non-Hispanic black respondents, and respondents of non-Hispanic other races. Use binary indicators *Hispanic*, *Black*, and *Other* to denote these race and ethnicity groups. Eq. 2 takes the following complete form.

3 $$Vaccinated_{i,t}=\beta_0\;+\beta_1X_{i,1}\;+\;\beta_2X_{i,\mathit2}\;+\;\beta_3PastVac_{i,t-\mathit1}\;+\beta_4PastFlu_{i,t-\mathit1}\;+\;\beta_{\mathit5\mathit.\mathit1}Hispanic_i\;+\;\beta_{5.2}Black_i\;+\;\beta_{5.3}Other_i\;+\;\beta_6PastVac_{i,t-\mathit1}\;\times\;PastFlu_{i,t-1}\;+\;\beta_{7.1}PastVac_{i,t-1}\;\times Hispanic_i\;+\;\beta_{7.2}PastVac_{i,t-1}\;\times Black_i\;+\;\beta_{7.3}PastVac_{i,t-1}\times Other_i\;+\;\beta_{8.1}PastFlu_{i,t-1}\;\times Hispanic_i\;+\;\beta_{8.2}PastFlu_{i,t-1}\times Black_i\;+\;\beta_{8.3}PastFlu_{i,t-1}\times Other_i\;+\;\beta_{9.1}PastVac_{i,t-1}\times PastFlu_{i,t-1}\;\times Hispanic_i\;+\;\beta_{9.2}PastVac_{i,t-1}\times PastFlu_{i,t-1}\;\times Black_i\;+\;\beta_{9.3}PastVac_{i,t-1}\times PastFlu_{i,t-1}\;\times Other_i\;+\;\varepsilon_i$$

The three-way interaction terms in Eq. 3 capture whether the relationship between a person’s influenza-related experiences and current vaccination varies across race/ethnicity groups. Equation specifications of other models are listed in Table 2.

The final data for analysis has 103,613 observations. To test the robustness of the results, logistic probability models were used, and the findings were similar. The empirical analysis used STATA 18 commands “svy: reg” and “svy: logit” to generate nationally representative results adjusted by the survey weights in MEPS.

## Results

Table 1 lists the summary statistics (percentage, standard deviation, and the number of observations) of the entire sample and samples by vaccination status in the first year. The last column is the *P* value from the t-test between the two groups by vaccination status.

**Table 1:** Summary statistics of the entire sample and by vaccination status in the first year

	Entire Sample	NOT Vac Year One^1^	Vac in Year One^2^	*P* value^3^
	% (SD) [n]^4^	% (SD) [n]	% (SD) [n]	
Vaccinated Year One	45.6(49.8)[48811]		100.0(0.0)[48811]	
Vaccinated Year Two	47.9(50.0)[51263]	15.8(36.5)[9233]	86.1(34.6)[42030]	0.00
Flu Infection Year One	5.2(22.1)[5520]	5.1(22.1)[2991]	5.2(22.2)[2529]	0.72
Always Insured	64.8(47.8)[69123]	54.2(49.8)[31417]	77.53(41.7)[37706]	0.00
Single Gap Insurance	3.8(19.1)[4021]	4.2(20.1)[2454]	3.22(17.7)[1567]	0.00
Transition Into Insured	7.7(26.6)[8175]	9.1(28.8)[5296]	5.92(23.6)[2879]	0.00
Transition Out Insured	5.2(22.1)[5505]	6.4(24.5)[3711]	3.69(18.9)[1794]	0.00
Temporary Insured	1.6(12.6)[1717]	2.1(14.2)[1198]	1.07(10.3)[519]	0.00
Repeatedly Insured	1.5(12.2)[1598]	1.8(13.2)[1030]	1.17(10.7)[568]	0.00
Always Insured	15.5(36.2)[16480]	22.2(41.6)[12882]	7.40(26.2)[3598]	0.00
Lack Access	43.9(49.6)[47048]	54.2(49.8)[31572]	31.7(46.5)[15476]	0.00
Non-Hispanic White	45.5(49.8)[48674]	40.8(49.2)[23776]	51.0(50.0)[24898]	0.00
Hispanic	26.5(44.1)[28400]	30.5(46.0)[17775]	21.8(41.3)[10625]	0.00
Non-Hispanic Black	18.8(39.0)[20079]	20.2(40.1)[11762]	17.0(37.6)[8317]	0.00
Non-Hispanic Other	9.3(29.0)[9939]	8.5(27.9)[4968]	10.2(30.2)[4971]	0.00
Age 18 to 25	14.7(35.4)[15770]	18.9(39.1)[10993]	9.8(29.7)[4777]	0.00
Age 26 to 35	18.6(38.9)[19901]	22.3(41.6)[12981]	14.2(34.9)[6920]	0.00
Age 36 to 45	18.4(38.8)[19709]	21.1(40.8)[12298]	15.2(35.9)[7411]	0.00
Age 46 to 55	18.8(39.1)[20164]	19.8(39.8)[11516]	17.7(38.2)[8648]	0.00
Age 56 to 64	13.6(34.2)[14511]	10.7(30.9)[6228]	17.0(37.5)[8283]	0.00
Age 65 and Above	15.9(36.6)[17037]	7.3(26.0)[4265]	26.2(44.0)[12772]	0.00
Male	45.6(49.8)[48827]	49.9(50.0)[29054]	40.5(49.1)[19773]	0.00
Female	54.4(49.8)[58265]	50.2(50.0)[29227]	59.5(49.1)[29038]	0.00
Less Than High School	22.5(41.7)[23947]	24.4(43.0)[14171]	20.1(40.1)[9776]	0.00
High School	29.9(45.8)[31893]	31.8(46.6)[18457]	27.7(44.7)[13436]	0.00
Less Than College	24.6(43.1)[26230]	24.4(43.0)[14162]	24.8(43.2)[12068]	0.11
College and More	23.0(42.1)[24521]	19.3(39.5)[11210]	27.4(44.6)[13311]	0.00
Excellent Health	57.8(49.4)[61845]	62.2(48.5)[36268]	52.4(49.9)[25577]	0.00
Good Health	29.8(45.8)[31947]	28.5(45.1)[16585]	31.5(46.4)[15362]	0.00
Fair or Poor Health	12.4(33.0)[13300]	9.3(29.1)[5428]	16.1(36.8)[7872]	0.00
Overweight or Obese	66.6(47.2)[70903]	65.4(47.6)[37873]	68.0(46.6)[33030]	0.00
Priority Condition	70.1(45.8)[75087]	62.1(48.5)[36165]	79.7(40.2)[38922]	0.00
Married	51.0(50.0)[54576]	47.8(50.0)[27861]	54.7(49.8)[26715]	0.00
Employed	49.9(50.0)[53482]	54.1(49.8)[31547]	44.9(49.7)[21935]	0.00
Student	8.3(27.6)[8879]	10.3(30.5)[6026]	5.8(23.5)[2853]	0.00
Low Income	40.7(49.1)[43594]	43.7(49.6)[25446]	37.2(48.3)[18148]	0.00
Middle Income	29.9(45.8)[31981]	30.8(46.2)[17943]	28.8(45.3)[14038]	0.00
High Income	29.4(45.6)[31517]	25.6(43.6)[14892]	34.1(47.4)[16625]	0.00
North East	15.8(36.4)[16875]	15.0(35.7)[8721]	16.7(37.3)[8154]	0.00
Midwest	19.4(39.5)[20737]	18.2(38.6)[10633]	20.7(40.5)[10104]	0.00
South	37.9(48.5)[40606]	39.4(48.9)[22972]	36.1(48.0)[17634]	0.00
West	27.0(44.4)[28874]	27.4(44.6)[15955]	26.5(44.1)[12919]	0.00
Panel 11	10.0(30.1)[10751]	11.6(32.0)[6729]	8.2(27.5)[4022]	0.00
Panel 12	7.6(26.5)[8125]	8.5(27.9)[4944]	6.5(24.7)[3181]	0.00
Panel 13	10.9(31.2)[11672]	12.1(32.6)[7066]	9.4(29.2)[4606]	0.00
Panel 14	9.9(29.9)[10635]	10.0(30.0)[5833]	9.8(29.8)[4802]	0.35
Panel 15	8.8(28.3)[9426]	8.7(28.2)[5068]	8.9(28.5)[4358]	0.18
Panel 16	11.3(31.7)[12122]	11.2(31.6)[6552]	11.4(31.8)[5570]	0.38
Panel 17	10.9(31.2)[11707]	10.7(30.9)[6249]	11.2(31.5)[5458]	0.02
Panel 18	10.2(30.2)[10899]	9.2(29.0)[5388]	11.3(31.7)[5511]	0.00
Panel 19	9.8(29.7)[10462]	8.8(28.3)[5123]	10.9(31.2)[5339]	0.00
Panel 20	10.6(30.7)[11293]	9.1(28.8)[5329]	12.2(32.8)[5964]	0.00

^1^Not vaccinated in the first year of survey

^2^Vaccinated in the first year of survey

^3^ *P* values from the t-tests between two groups by vaccination status in the first year

^4^% (SD) [n] = percentage, standard deviation, and the number of observations

Among people who were not vaccinated in the first year, i.e., those in column two, 15.8% got vaccinated in the second year. The vaccination rate was 86.1% among people who were vaccinated in the first year in column three. This large difference suggests that first-year vaccination status is a strong predictor for people’s subsequent vaccination.

Significant differences exist in demographics, socioeconomic factors, and health-related variables by vaccination status. For example, the vaccinated population had a higher percentage of being always insured (77.5% vs 54.2% among people who were unvaccinated), a smaller percentage reporting a lack of access to healthcare resources (31.7% vs 54.2% among the unvaccinated population), a higher proportion of the elderly population (26.2% vs 7.2%), and a higher level of education in college or more (27.4% vs 19.3%). People who were non-Hispanic white were more likely to get vaccinated than people who were Hispanic, non-Hispanic black, and other non-Hispanic races.

The vaccinated population had lower health than the unvaccinated population. For example, among the vaccinated people, 16.13% reported fair or poor health, 68.01% reported overweight or obese, and 79.74% had priority conditions. In contrast, the percentages for the unvaccinated population were 9.31% with fair or poor health, 65.39% overweight or obese, and 62.05% with priority conditions. The vaccinated group had 34.06% in the high-income category, higher than the 25.55% for the unvaccinated group. These observations are consistent with previous findings on factors related to flu vaccination decisions. The summary statistics of the vaccinated population in the second year were very close to those in the first year. For conciseness, they were not reported.

Table 2 lists the results from different models. To be concise, it only reports coefficients for past flu vaccination, past flu infection, race/ethnicity, income, education, health status, age, and interaction terms. All other socioeconomic, demographic, healthcare variables and year-fixed effects are included in the regressions, though not reported in Table 2. All regressions are weighted by MEPS sample weights.

**Table 2:** Coefficients and standard errors from linear probability models

	(1)^a^		(2)^b^		(3)^c^
Vaccinated	0.684*** (0.004)	Vaccinated	0.718*** (0.004)	Vaccinated	0.630*** (0.005)
Infected	0.021** (0.009)	Infected	0.044*** (0.012)	Infected	0.014 (0.016)
Vaccinated x Infected	−0.023* (0.014)	Vaccinated x Infected	−0.054*** (0.017)	Vaccinated x Infected	−0.010 (0.024)
		Hispanic	0.067*** (0.006)	Middle Income	−0.031*** (0.005)
		Non-Hispanic Black	0.024*** (0.006)	High Income	−0.037*** (0.005)
		Non-Hispanic Other Race	0.065*** (0.010)	Vaccinated x Middle Income	0.056*** (0.007)
		Vaccinated x Hispanic	−0.129*** (0.008)	Vaccinated x High Income	0.090*** (0.007)
		Vaccinated x Non-Hispanic Black	−0.094*** (0.009)	Infected x Middle Income	−0.008 (0.023)
		Vaccinated x Non-Hispanic Other	−0.083*** (0.012)	Infected x High Income	0.029 (0.024)
		Infected x Hispanic	−0.083*** (0.020)	Vaccinated x Infected x Middle Income	0.011 (0.034)
		Infected x Non-Hispanic Black	0.006 (0.031)	Vaccinated x Infected x High Income	−0.044 (0.034)
		Infected x Non-Hispanic Other	−0.094*** (0.035)		
		Vaccinated x Infected x Hispanic	0.097*** (0.029)		
		Vaccinated x Infected x Non-Hispanic Black	0.044 (0.045)		
		Vaccinated x Infected x Non-Hispanic Other	0.133*** (0.044)		
Observations	103613		103613		103613
R-squared	0.55		0.55		0.55
	(4)^d^		(5)^e^		(6)^f^
Vaccinated	0.672*** (0.004)	Vaccinated	0.663*** (0.009)	Vaccinated	0.672*** (0.004)
Infected	0.019* (0.010)	Infected	−0.044 (0.030)	Infected	0.018* (0.009)
Vaccinated x Infected	−0.024 (0.016)	Vaccinated x Infected	0.029 (0.036)	Vaccinated x Infected	−0.021 (0.016)
College or More	0.014*** (0.005)	Good Health	−0.041*** (0.009)	Age 65 or Above	−0.004 (0.009)
Vaccinated x College or More	0.038*** (0.007)	Very Good or Excellent Health	−0.060*** (0.009)	Vaccinated x Age 65 or Above	0.083*** (0.009)
Infected x College or More	0.009 (0.024)	Vaccinated x Good Health	0.020** (0.009)	Infected x Age 65 or Above	0.011 (0.056)
Vaccinated x Infected x College or More	−0.002 (0.030)	Vaccinated x Very Good or Excellent Health	0.024** (0.009)	Vaccinated x Infected x Age 65 or Above	0.004 (0.057)
		Infected x Good Health	0.080** (0.036)		
		Infected x Very Good or Excellent Health	0.067** (0.032)		
		Vaccinated x Infected x Good Health	−0.066 (0.042)		
		Vaccinated x Infected x Very Good or Excellent Health	−0.050 (0.038)		
Observations	103613		103613		103613
R-squared	0.55		0.55		0.55

Standard errors are in parentheses

All models control for demographic, socioeconomic, and health-related variables

Some of them are not reported here

Full results are available upon request

* *p* < 0.10, ** *p* < 0.05, *** *p* < 0.01

^a^ Model in column 1:

*Vaccinated*_*i,t*_ *= β*_*0*_ *+ β*_*1*_*X*_*i,1*_ *+ β*_*2*_*X*_*i,2*_ *+ β*_*3*_*PastVac*_*i,t−1*_ *+ β*_*4*_*PastFlu*_*i,t−1*_ *+ β*_*5*_*PastVac*_*i,t−1*_*×PastFlu*_*i,t−1*_ *+ ε*_*i*_

*Vaccinated*_*i,t*_ is a dummy variable that equals one if individual *i* has been vaccinated at time *t*. *X*_*i,1*_ is the set of respondents’ individual and family characteristics; *X*_*i,2*_ is the set of the year and quarterly indicators to capture the flu season’s severity. *PastVac*_*i,t−1*_ indicates individual *i* vaccination status in year *t-1*. It takes the value of one if individual *i* was vaccinated in year *t-1*. *PastFlu*_*i,t−1*_ indicates individual *i* influenza status in year *t-1*. It takes the value of one if the individual *i* had influenza in year *t-1*

^b^ Model in column 2:

*Vaccinated*_*i,t*_*=β*_*0*_ *+ β*_*1*_*X*_*i,1*_ *+ β*_*2*_*X*_*i,2*_ *+ β*_*3*_*PastVac*_*i,t-1*_ *+ β*_*4*_*PastFlu*_*i,t-1*_ *+ β*_*5.1*_*Hispanic*_*i*_ *+ β*_*5.2*_ *Black*_*i*_ *+ β*_*5.3*_*Other*_*i*_*+ β*_*6*_*PastVac*_*i,t-1*_*×PastFlu*_*i,t-1*_ *+ β*_*7.1*_*PastVac*_*i,t-1*_*×Hispanic*_*i*_ *+ β*_*7.2*_*PastVac*_*i,t-1*_*×Black*_*i*_ *+ β*_*7.3*_*PastVac*_*i,t-1*_*×Other*_*i*_*+ β*_*8.1*_*PastFlu*_*i,t-1*_*×Hispanic*_*i*_ *+ β*_*8.2*_*PastFlu*_*i,t-1*_*×Black*_*i*_ *+ β*_*8.3*_*PastFlu*_*i,t-1*_*×Other*_*i*_ *+ β*_*9.1*_*PastVac*_*i,t-1*_*×PastFlu*_*i,t-1*_*×Hispanic*_*i*_*+β*_*9.2*_*PastVac*_*i,t-1*_*×PastFlu*_*i,t-1*_*×Black*_*i*_ *+ β*_*9.3*_*PastVac*_*i,t-1*_*×PastFlu*_*i,t-1*_*×Other*_*i*_ + *ε*_*i*_

In addition to variables in model 1, model 2 adds a three-way interaction term with a person’s race/ethnicity

^c^ Model in column 3:

*Vaccinated*_*i,t*_ *= β*_*0*_ *+ β*_*1*_*X*_*i,1*_ *+ β*_*2*_*X*_*i,2*_ *+ β*_*3*_*PastVac*_*i,t-1*_ *+ β*_*4*_*PastFlu*_*i,t-1*_ *+ β*_*5.1*_*Middle Income*_*i*_ *+ β*_*5.2*_*High Income*_*i*_*+β*_*6*_*PastVac*_*i,t-1*_*×PastFlu*_*i,t-1*_ *+ β*_*7.1*_*PastVac*_*i,t-1*_*×Middle Income*_*i*_ *+ β*_*7.2*_*PastVac*_*i,t-1*_*×High Income*_*i*_*+ β*_*8.1*_*PastFlu*_*i,t-1*_*×Middle Income*_*i*_ *+ β*_*8.2*_*PastFlu*_*i,t-1*_*×High Income*_*i*_ *+ β*_*9.1*_*PastVac*_*i,t-1*_*×PastFlu*_*i,t-1*_*×Middle Income*_*i*_*+β*_*9.2*_*PastVac*_*i,t-1*_*×PastFlu*_*i,t-1*_*×High Income*_*i*_ *+ ε*_*i*_

In addition to variables in model 1, model 3 adds a three-way interaction term with a person’s income

^d^ Model in column 4:

*Vaccinated*_*i,t*_ *= β*_*0*_ *+ β*_*1*_*X*_*i,1*_ *+ β*_*2*_*X*_*i,2*_ *+ β*_*3*_*PastVac*_*i,t-1*_ *+ β*_*4*_*PastFlu*_*i,t-1*_ *+ β*_*5*_ *College or More*_*i*_ *+ β*_*6*_*PastVac*_*i,t-1*_*×PastFlu*_*i,t-1*_*+ β*_*7*_*PastVac*_*i,t-1*_*×College or More*_*i*_ *+ β*_*8*_*PastFlu*_*i,t-1*_*×College or More*_*i*_ *+ β*_*9*_*PastVac*_*i,t-1*_*×PastFlu*_*i,t-1*_*×College or More*_*i*_ *+ ε*_*i*_

In addition to variables in model 1, model 4 adds a three-way interaction term with a person’s education

^e^ Model in column 5:

*Vaccinated*_*i,t*_ *= β*_*0*_ *+ β*_*1*_*X*_*i,1*_ *+ β*_*2*_*X*_*i,2*_ *+ β*_*3*_*PastVac*_*i,t-1*_ *+ β*_*4*_*PastFlu*_*i,t-1*_ *+ β*_*5.1*_*Good Health*_*i*_ *+ β*_*5.2*_*Very Good or Excellent Health*_*i*_*+β*_*6*_*PastVac*_*i,t-1*_*×PastFlu*_*i,t-1*_ *+ β*_*7.1*_*PastVac*_*i,t-1*_*×Good Health*_*i*_ *+ β*_*7.2*_*PastVac*_*i,t-1*_*×Very Good or Excellent Health*_*i*_*+β*_*8.1*_*PastFlu*_*i,t-1*_*×Good Health*_*i*_ *+ β*_*8.2*_*PastFlu*_*i,t-1*_*×Very Good or Excellent Health*_*i*_*+β*_*9.1*_*PastVac*_*i,t-1*_*×PastFlu*_*i,t-1*_*×Good Health*_*i*_ *+ β*_*9.2*_*PastVac*_*i,t-1*_*×PastFlu*_*i,t-1*_*×Very Good or Excellent Health*_*i*_ *+ ε*_*i*_

In addition to variables in model 1, model 5 adds a three-way interaction term with a person’s health

^f^ Model in column 6:

*Vaccinated*_*i,t*_ *= β*_*0*_ *+ β*_*1*_*X*_*i,1*_ *+ β*_*2*_*X*_*i,2*_ *+ β*_*3*_*PastVac*_*i,t-1*_ *+ β*_*4*_*PastFlu*_*i,t-1*_ *+ β*_*5*_ *Age 65 or Above*_*i*_ *+ β*_*6*_*PastVac*_*i,t-1*_*×PastFlu*_*i,t-1*_*+ β*_*7*_*PastVac*_*i,t-1*_*×Age 65 or Above*_*i*_ *+ β*_*8*_*PastFlu*_*i,t-1*_*×Age 65 or Above*_*i*_ *+ β*_*9*_*PastVac*_*i,t-1*_*×PastFlu*_*i,t-1*_*×Age 65 or Above*_*i*_ *+ ε*_*i*_

In addition to variables in model 1, model 6 adds a three-way interaction term with a person’s age

Column one in Table 2 lists output from Eq. 1, i.e., the model that includes the binary indicator for vaccination in the first year (*Vaccinated* in the table), flu infection in the first year (*Infected* in the table), and their interaction term (*Vaccinated X Infected*). Both first-year vaccination and infection are associated with a higher probability of vaccination in the following year.

However, the first-year vaccination has a larger effect on the second-year vaccination than the first-year infection. The former is associated with an increase of 68.4% in the second-year vaccination probability, while the latter is associated with a rise of 2.1%. The coefficient of the interaction term, *Vaccinated X Infected*, is −2.3%, suggesting that previous infection history will reduce the impact of past vaccination by 2.3% from 68.4% to 66.1%. However, the interaction term’s coefficient, −2.3%, is much smaller than that of first-year vaccination, 68.4%, implying that although previous infection history lowers the impact of prior vaccination, the influence is small.

Columns two to six list results with three-way interaction terms among flu-related experiences and other factors, i.e., Eq. 2. For example, column two lists estimates in Eq. 3. It includes first-year vaccination, infection, and their interaction term. In addition, they interact with race/ethnicity indicators. The purpose is to examine whether the relationship varies across these race/ethnicity groups. The other columns are arranged similarly. Detailed model specifications are at the bottom of Table 2.

Table 3 lists the predicted probabilities and their 95% confidence intervals based on coefficients from Table 2 to facilitate the interpretation of the three-way interaction terms. Comparisons of these probabilities are more straightforward to examine whether the impacts of flu-related experiences vary across various demographic, socioeconomic, or health status groups.

**Table 3:** Predicted second-year vaccination probability by demographic, socioeconomic, and health variables

Vac & Infection Status	Pop. Group	Probability in Pct.	Vac & Infection Status	Pop. Group	Probability in Pct.
Not Vac, Not Infected	Overall	21.2 [20.5, 22.0]	Not Vac, Not Infected	Less Than College	20.1 [19.3, 20.8]
Not Vac, Infected		23.4 [21.5, 25.2]	Not Vac, Infected		22.0 [20.0, 24.1]
Vac, Not Infected		89.6 [89.0, 90.2]	Vac, Not Infected		87.3 [86.7, 87.9]
Vac and Infected		89.4 [87.6, 91.3]	Vac and Infected		86.8 [84.5, 89.2]
Not Vac, Not Infected	White	18.9 [18.0, 19.7]	Not Vac, Not Infected	College or More	21.5 [20.4, 22.7]
Not Vac, Infected		23.3 [20.7, 25.8]	Not Vac, Infected		24.3 [20.2, 28.4]
Vac, Not Infected		90.7 [90.1, 91.3]	Vac, Not Infected		92.5 [91.8, 93.3]
Vac and Infected		89.6 [87.4, 91.8]	Vac and Infected		92.8 [90.3, 95.2]
Not Vac, Not Infected	Hispanic	25.6 [24.6, 26.6]	Not Vac, Not Infected	Poor or Fair Health	25.0 [23.2, 26.8]
Not Vac, Infected		21.7 [18.7, 24.6]	Not Vac, Infected		20.6 [15.1, 26.2]
Vac, Not Infected		84.5 [83.2, 85.8]	Vac, Not Infected		91.3 [90.3, 92.3]
Vac and Infected		84.9 [81.1, 88.7]	Vac and Infected		89.8 [86.2, 93.5]
Not Vac, Not Infected	Black	21.3 [20.1, 22.4]	Not Vac, Not Infected	Good Health	20.9 [20.0, 21.8]
Not Vac, Infected		26.3 [20.9, 31.6]	Not Vac, Infected		24.5 [20.8, 28.3]
Vac, Not Infected		83.7 [82.6, 84.9]	Vac, Not Infected		89.2 [88.5, 89.9]
Vac and Infected		87.6 [82.0, 93.3]	Vac and Infected		89.1 [86.4, 91.9]
Not Vac, Not Infected	Other	25.4 [23.4, 27.4]	Not Vac, Not Infected	Very Good or Excellent	19.0 [18.2, 19.9]
Not Vac, Infected		20.3 [14.1, 26.5]	Not Vac, Infected		21.3 [19.0, 23.7]
Vac, Not Infected		88.9 [87.6, 90.3]	Vac, Not Infected		87.8 [87.1, 88.4]
Vac and Infected		91.7 [87.1, 96.3]	Vac and Infected		87.9 [85.3, 90.4]
Not Vac, Not Infected	Low Income	23.7 [22.8, 24.6]	Not Vac, Not Infected	Less Than Age 65	19.0 [18.3, 19.6]
Not Vac, Infected		25.0 [21.9, 28.2]	Not Vac, Infected		20.8 [18.9, 22.7]
Vac, Not Infected		86.7 [85.8, 87.6]	Vac, Not Infected		86.1 [85.5, 86.8]
Vac and Infected		87.0 [83.6, 90.5]	Vac and Infected		85.8 [83.6, 88.1]
Not Vac, Not Infected	Middle Income	20.5 [19.5, 21.6]	Not Vac, Not Infected	Age 65 or Above	18.5 [16.6, 20.4]
Not Vac, Infected		21.1 [18.0, 24.2]	Not Vac, Infected		21.4 [10.8, 32.0]
Vac, Not Infected		89.1 [88.3, 90.0]	Vac, Not Infected		94.0 [93.3, 94.8]
Vac and Infected		89.8 [86.6, 93.0]	Vac and Infected		95.3 [93.9, 96.6]
Not Vac, Not Infected	High Income	20.0 [19.0, 20.9]			
Not Vac, Infected		24.2 [20.8, 27.6]			
Vac, Not Infected		92.0 [91.2, 92.7]			
Vac and Infected		90.8 [88.1, 93.4]			

*Not Vac, Not Infected* Not Vaccinated and Not Infected in the first year, *Not Vac, Infected* Not Vaccinated and Infected in the first year, *Vac, Not Infected* Vaccinated and Not Infected in the first year; *Vac and Infected* Vaccinated and Infected in the first year

An interesting finding in this table is that both the lowest and highest probability are in the population aged 65 and above. Among respondents aged 65 and above, the predicted probability is 18.5% (95% CI: 16.6, 20.4) for those who are not vaccinated and not infected in the first year. In contrast, among respondents who are vaccinated and infected in the previous year, the predicted probability is 95.3% (95% CI: 93.9, 96.6).

Of the entire sample of the unvaccinated population, the 95% confidence intervals overlap between those who are not infected (21.2%; 95% CI:20.5, 22.0) and those who are infected (23.4%; 95% CI:21.5, 25.2). Similarly, among the entire sample of the first-year vaccinated population groups, the 95% confidence intervals overlap between first-year uninfected respondents (89.6%; 95% CI:0.89.0, 90.2) and first-year infected respondents (89.4%; 95% CI: 87.6, 91.3). This result is consistent with column one in Table 2 because the coefficient for the interaction term between vaccination and infection is only significant at the 10% level.

In the four race and ethnicity groups, the lowest predicted probability of vaccination in the second year is 18.9% (95% CI: 18.0, 19.7) among unvaccinated and uninfected people who are non-Hispanic white. The highest probability of second-year vaccination is 91.7% (95% CI: 87.1, 96.3) among vaccinated and infected populations of non-Hispanic other races. The only significant relationship of previous influenza infection is among the unvaccinated respondents who are non-Hispanic white. The predicted probability of getting vaccinated in the second year for the infected individuals 23.3% (95% CI: 20.7, 25.8) is significantly higher than the uninfected individuals 18.9% (95% CI:18.0, 19.7) because the 95% confidence intervals do not overlap.

Except for respondents who are non-Hispanic white, the highest predicted probabilities of vaccination in the second year are among those who are vaccinated and infected, including 84.9% (95% CI: 81.1, 87.7) for the Hispanic population, 87.6% (95% CI: 82.0, 93.3) for respondents who are non-Hispanic black, and 91.7% (95% CI: 87.1, 96.3) for respondents of non-Hispanic other races. The highest predicted probability among respondents of non-Hispanic white is for the vaccinated and uninfected individuals: 90.7% (95% CI: 90.1, 91.3). However, within each race and ethnicity group, the probabilities among the population that is vaccinated and infected are not significantly different from the population that is vaccinated and uninfected.

In the three income groups, the highest probability is 92.0% (95% CI: 91.2, 92.7) for the high-income group who are vaccinated and not infected. Influenza infection’s impact is insignificant among the vaccinated populations or unvaccinated populations within each income group.

Comparison between the two education levels shows that people with college or higher levels of education who are vaccinated and infected have the highest probability at 92.8% (95% CI:90.3, 95.2). Among the three health status groups, the highest probability, 91.3% (95% CI:90.3, 92.3), is for the vaccinated and uninfected people with poor or fair health.

**Fig. 1:**
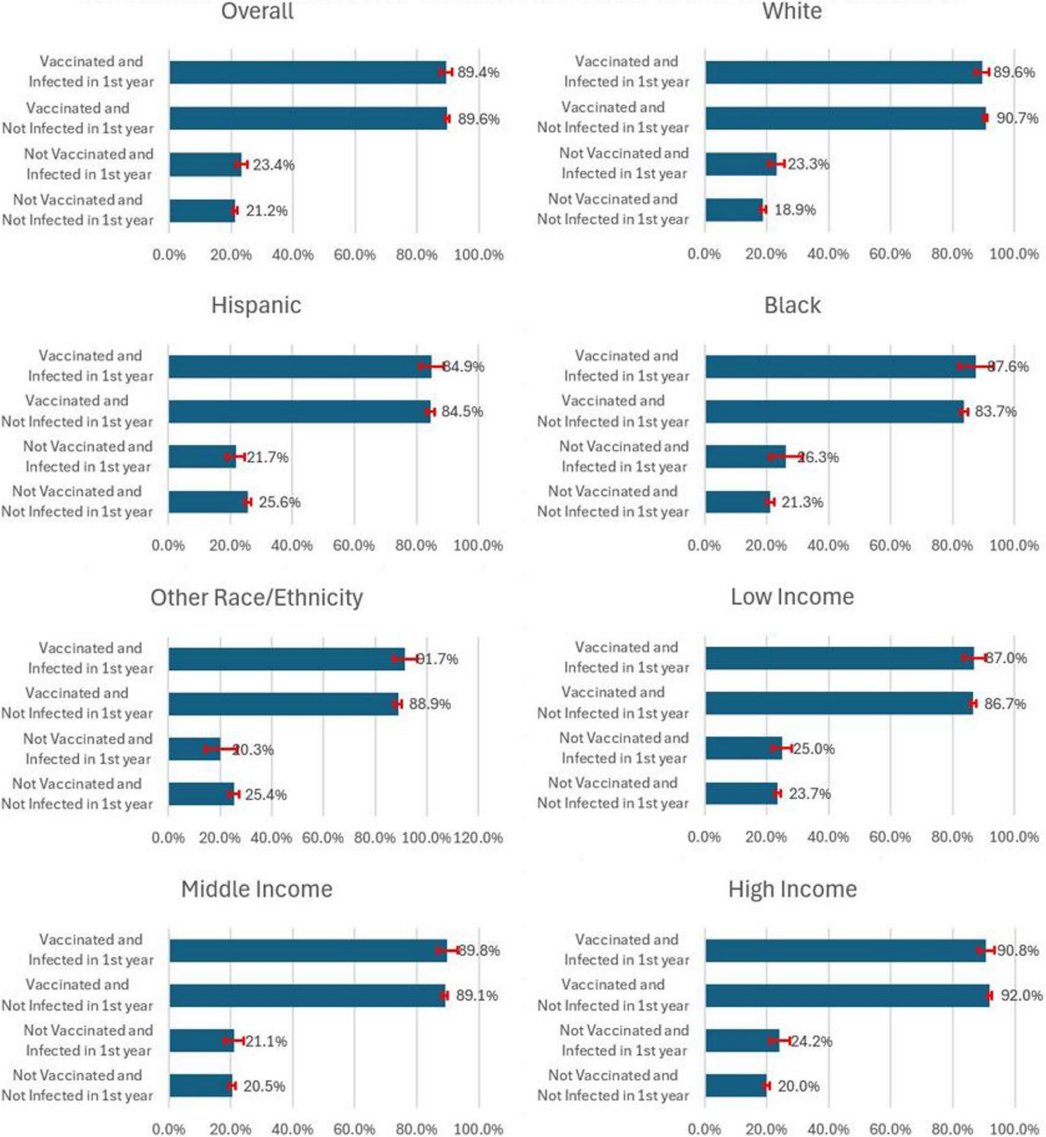
Predicted second-year vaccination probability by race, ethnicity, and income

**Fig. 2:**
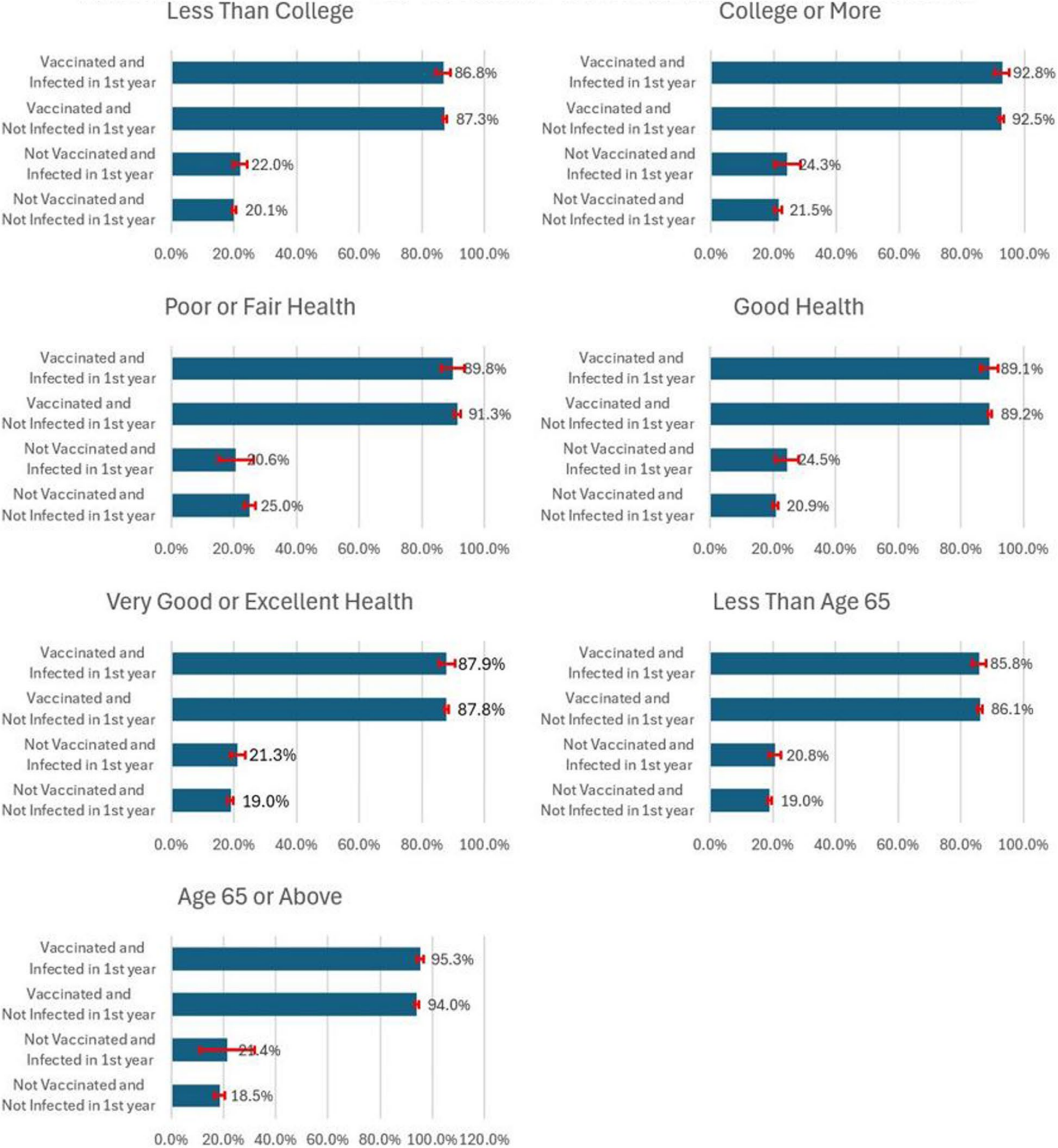
Predicted second-year vaccination probability by education, health, and age

Previous comparisons are displayed in Figs. 1 and 2. Figure 1 shows the probability for the entire population and subgroups by race/ethnicity and income levels. Figure 2 displays the probability by education, health levels, and age groups.

Appendix A lists relative probability changes compared to the reference group: people who were not vaccinated and not infected in the past. This comparison is another way to interpret coefficients from Table 2. To test the robustness of the results, all regressions and predicted probabilities are estimated using logistic probability models. The results are listed in the appendix. The findings are very close to those from linear probability models.

## Discussion

The empirical findings suggest that a person’s previous vaccination history is the strongest predictor of their future vaccination. This finding is consistent with other empirical studies [11–14, 16, 22]. For instance, in studies using data from England and Taiwan [12 and 14], the authors found previous vaccination history to be the most important factor associated with future vaccination decisions. Similarly, a systematic review of 470 articles from 2005 to 2016 found the past vaccination to be one of the most important determinants for future vaccinations [17].

Another finding of this study is that previous infection is not a significant predictor, especially considering demographic, socioeconomic, and health-related factors. Earlier work had similar findings. For example, an analysis of 1,005 adults in the U.S. [23] found that the experience of influenza illness only had a relatively small association with influenza vaccination compared to other factors such as age, race/ethnicity, and access to healthcare providers. Three studies [11, 12, 14] examined factors influencing parents’ vaccination decisions for their children. Although some parents listed the past influenza experience as one of the reasons, it was not among the most important ones. In another study, the authors found that past influenza pandemics were not associated with post-pandemic vaccination [16].

Previous work proposed some explanations for this somewhat counter-intuitive finding. One study [22] suggested that people who had influenza in the past might perceive the illness they experienced as mild. Therefore, they would consider the vaccine less necessary because of its lowered benefit. The authors further suggested that the participants might not distinguish between influenza and other flu-like illnesses. If they experienced these other illnesses and considered them as mild, they would also lower the perceived benefit from vaccination. Another study proposed similar explanations. The authors found that previous infection was not a significant determinant. “Many adults appear to consider influenza to be either an unlikely health threat or one that would be ‘manageable’ (i.e., would not cause serious illness) [23].” Finally, one study suggested that infected people believed the infection already gave them immunity to the same strain the vaccines protected against [24].

Like previous studies, there are significant differences in the predicted vaccination probability by demographic, socioeconomic, and health variables. For example, among the population vaccinated and not infected in the first year, the highest probability of vaccination in the second year, 90.7% (95% CI: 90.1, 91.3), is among people who are non-Hispanic white, and it is significantly higher than the people who are Hispanic (84.5%; 95% CI: 83.2, 85.8) and people who are non-Hispanic black (83.7%; 95% CI: 82.6, 84.9). Disparities are also found within different income groups, education levels, health status, and age groups.

The previously unvaccinated population could be the focus of future vaccination efforts because they have much lower predicted vaccination probabilities. Another group that needs attention is people aged 65 and above. As the most vulnerable age group, they exhibit the largest disparity based on their previous vaccination and infection. Consistent with the meta-analysis of CDC-sponsored studies [5], previously vaccinated and infected elderly have the highest vaccination rates, while unvaccinated and uninfected ones have the lowest rates. We need better ways to motivate the previously unvaccinated and uninfected elderly population [25].

Recent studies suggest that differences in Covid-19 vaccination rates mirror flu vaccination rates due to the same concerns and attitudes [9] and that the receipt of the influenza vaccine is associated with lower Covid susceptibility and severity [26]. It is worth noting that the relationships observed in this study may have changed in recent years (i.e., after the study period), especially in light of the increased polarization surrounding vaccination status observed during and after the COVID-19 pandemic. Although several studies reported that the COVID-19 pandemic has increased influenza vaccination rates [27–29], discrepancies across age and different population groups remained and could have increased.

For example, the CDC report [29] showed that the increase came from adults aged 18 and older, while vaccination rates decreased among children under four. People who were white or Asian were more likely to get vaccinated than other ethnic groups [28]. Previous vaccination history was still one of the main determinants and predictors for current and future influenza vaccinations. One study found that consistently vaccinated people in the last five seasons were 83.7% more likely to get vaccinated in the subsequent seasons [28].

Another important lesson from the Covid pandemic is the role (and risk) of mainstream media and social media in facilitating more effective outreach. Social media can amplify and expedite the spread of misinformation and anti-vaccine campaigns that are directly linked with delayed and refused vaccinations [30–32]. These adverse effects of social media on reducing vacations and damaging trust in reliable information increased during COVID-19, especially among the minority population [31, 33]. Governments and public health agencies developed various strategies to use mainstream and social media to debunk misinformation, warn people about unreliable messages, and deliver evidence-based materials [30, 32]. However, reviews of these approaches showed no or minimal effects on vaccination uptake [30, 34].

Although there are no clear guidelines on the effective use of social media to promote vaccinations, governments and public health agencies can follow some insights during the pandemic and expert recommendations [30, 31, 33, 34]. Some insights and recommendations include tailoring the content and format of messages to address the targeted audience’s concerns and knowledge, expansions beyond the benefits and risks based on probabilities, strengthening the relationship between public health departments and mainstream and social media platforms, and choosing the correct messengers, champions, and providers considered trustworthy to the public and local communities [30, 31, 33, 34].

To summarize, it is worthwhile to examine whether and how patterns in this study have changed in the post-Covid era and the role of social media in facilitating vaccination outreach.

This paper has limitations. First, the results should be interpreted as association and not causality, mainly due to missing variable bias. Although the models have controlled a large set of demographics, socioeconomic, and health variables, it is possible that some unobservable factors are not accounted for, contributing to the heterogeneity between vaccinated and unvaccinated groups. Although there is no contemporaneous relationship, it is possible that a person’s past vaccination, infection history, and current vaccination can be affected by the same unobservable factors. Thus, the estimated coefficients could be biased.

Second, the medical and vaccination conditions are self-reported. Although MEPS validated a sample of the responses through medical providers, it remains possible that the responses are subject to recall accuracy. Some respondents might incorrectly report previous infections or vaccinations.

Third, because of data limitations in MEPS, this study did not examine citizenship or place of birth, even though people of different immigration statuses and nativity have different uptakes of vaccines [35].

In conclusion, this study provides empirical evidence of the importance of previous vaccination history. It gives directions on the population groups that need more attention and efforts to improve vaccination rates.

## Supplementary Information

Supplementary Material 1.

## Data Availability

The datasets generated and analyzed during the current study are publicly available in the Medical Expenditure Panel Survey (MEPS): https://meps.ahrq.gov/mepsweb/ MEPS is a set of large-scale surveys of families and individuals, their medical providers, and employers across the United States.

## Footnotes

1 MEPS full-year longitudinal file is available at https://meps.ahrq.gov/mepsweb/data_stats/download_data_files_results.jsp?cboDataYear=All&cboDataTypeY=1%2CHousehold+Full+Year+File&buttonYearandDataType=Search&cboPufNumber=All&SearchTitle=Longitudinal Medical conditions file is available at https://meps.ahrq.gov/mepsweb/data_stats/download_data_files_results.jsp?cboDataYear=All&cboDataTypeY=1%2CHousehold+Full+Year+File&buttonYearandDataType=Search&cboPufNumber=All&SearchTitle=Medical+Conditions
