# Supplementary material for: Relationship between influenza-related experience and current vaccination outcome
Source: BMC Public Health. 2025 Jan 16;25:174. doi: 10.1186/s12889-024-21263-5 (PMC11736949; doi:10.1186/s12889-024-21263-5)
Supplement: Supplementary file 1 — Supplementary Material 1. [file 12889_2024_21263_MOESM1_ESM.docx]

**Appendix A**

**Detailed Model Specifications, Alternative Interpretations, and Robustness Tests**

**Detailed Model Specifications**

Two sets of linear probability models were estimated to analyze the relationship between influenza-related experiences and vaccination outcomes and how this relationship varies across demographic and socioeconomic factors. The first model includes a person’s previous vaccination, infection, and interaction term. It has the following specification:

*Vaccinated_i,t_=β_0_ +β_1_X_i,1_+β_2_X_i,2_+β_3_PastVac_i,t-1_ + β_4_PastFlu_i,t-1_ + β_5_PastVac_i,t-1_×PastFlu_i,t-1_ +ε_i_ --- (1)*

Where *Vaccinated_i,t_* is a dummy variable that equals one if individual *i* has been vaccinated at time *t*. *X_i,1_* is the set of respondents’ individual and family characteristics; *X_i,2_* is the set of the year and quarterly indicators to capture the flu season’s severity.

*PastVac_i,t-1_* indicates individual *i* vaccination status in year *t-1*. It takes the value of one if individual *i* was vaccinated in year *t-1*. *PastFlu_i,t-1_* is an indicator on individual *i* influenza status in year *t-1*. It takes the value of one if the individual *i* had influenza in year *t-1*.

A person’s vaccination and flu status from the previous year could influence each other. To capture this effect, we add the interaction term, *PastVac_i,t-1_×PastFlu_i,t-1_* The coefficient, *β_5_* for the interaction term, captures whether past influenza infection affects the impact of past vaccination on current vaccination status. *β_3_, β_4_* and *β_5_* capture four types of flu-related experience in the previous year:

| Individual Types | Predicted Probability |
| --- | --- |
| Not vaccinated, and not infected by flu (reference group) | *β_0_ +β_1_X_i,1_+β_2_X_i,2_* |
| Vaccinated, and infected by flu | *β_0_+β_1_X_i,1_+β_2_X_i,2_+β_3_+β_4_+β_5_* |
| Vaccinated, and not infected by flu | *β_0_+β_1_X_i,1_+β_2_X_i,2_+β_3_* |
| Not vaccinated, and infected by flu | *β_0_+β_1_X_i,1_+β_2_X_i,2_+β_4_* |

Depending on the coefficients, *β_3_, β_4_* and *β_5_* and their significance, I can tell how individuals’ experience in flu vacation and flu infection is related to their recent decision to get vaccinated. The four types of individuals correspond to loss aversion and framing in behavioral economics. In behavioral economics, people react more to losses than the same amount of gain, and messages framed in losses are more effective than gains.^1^ For example, a weight loss program with a monetary penalty is more effective than awards, even though people end up with the same amount of money when they fail (or succeed) in the program. Similarly, a negative experience with flu shots is more memorial and salient than a positive experience and could cause one to overestimate the risk of vaccination.^2^

The relationship in equation 1 can vary across demographic and socioeconomic factors. To capture these changes, I include interactions between flu-related experience with individual-level factors in equation 2.

*Vaccinated_i,t_ =β_0_ +β_1_X_i,1_ +β_2_X_i,2_ +β_3_PastVac_i,t-1_ +β_4_PastFlu_i,t-1_+ β_5_Z_i_ + β_6_PastVac_i,t-1_×PastFlu_i,t-1_ + β_7_PastVac_i,t-1_×Z_i_ + β_8_Z_i_×PastFlu_i,t-1_ + β_9_PastVac_i,t-1_×PastFlu_i,t-1_×Z_i_*+ *ε_i_ --- (2)*

where *Z_i_* is one of a person’s demographic and socioeconomic factors, including race/ethnicity, income, education, health status, and elderly status. Depending on which of the five factors are used in equation 2, there will be a total of five models to be estimated.

Take race/ethnicity as an example. There are four race/ethnicity categories in this study: non-Hispanic white (reference group), Hispanic, non-Hispanic black, and non-Hispanic other races. Use *Hispanic*, *Black,* and *Other* to denote the binary indicators for Hispanic, non-Hispanic black, and non-Hispanic other races. Equation 2 takes the following full form.

*Vaccinated_i,t_=β_0_ + β_1_X_i,1_ + β_2_X_i,2_ + β_3_PastVac_i,t-1_ + β_4_PastFlu_i,t-1_ + β_5.1_Hispanic_i_ + β_5.2_Black_i_ + β_5.3_Other_i_+*

*β_6_PastVac_i,t-1_×PastFlu_i,t-1_ + β_7.1_PastVac_i,t-1_×Hispanic_i_ + β_8.1_Hispanic_i_×PastFlu_i,t-1_ + β_7.2_PastVac_i,t-1_×Black_i_ +*

*β_8.2_ Black_i_×PastFlu_i,t-1_ + β_7.3_PastVac_i,t-1_×Other_i_ + β_8.3_ Other_i_×PastFlu_i,t-1_+ β_9.1_PastVac_i,t-1_×PastFlu_i,t-1_×Hispanic_i_ + β_9.2_PastVac_i,t-1_×PastFlu_i,t-1_×Black_i_ + β_9.3_PastVac_i,t-1_×PastFlu_i,t-1_×Other_i_* + *ε_i_ --- (3)*

The coefficients, *β_3_* to *β_9.3,_* and their combinations will predict 16 different probabilities for vaccination depending on a person’s past vaccination, flu status, and race/ethnicity categories. The following are the 16 different probabilities (non-Hispanic white, not vaccinated, and not infected as the reference group):

| Individual Types | Predicted Probability |
| --- | --- |
| White, not vaccinated, and not infected (reference) | *β_0_+ β_0_+β_1_X_i,1_+β_2_X_i,2_* |
| White, vaccinated, and infected | *β_0_+ β_0_+β_1_X_i,1_+β_2_X_i,2_+β_3_+β_4_+β_6_* |
| White, vaccinated, and not infected | *β_0_+ β_0_+ β_1_X_i,1_+β_2_X_i,2_+β_3_* |
| White, not vaccinated, and infected | *β_0_+β_1_X_i,1_+β_2_X_i,2_+β_4_* |
|  |  |
| Hispanic, vaccinated, and infected | *β_0_+ β_0_+β_1_X_i,1_+β_2_X_i,2_+β_3_+β_4_+β_5.1_+β_6_+β_7.1_+β_8.1_+β_9.1_* |
| Hispanic, vaccinated, and not infected | *β_0_+ β_0_+β_1_X_i,1_+β_2_X_i,2_+β_3_+β_5.1_+β_7.1_* |
| Hispanic, not vaccinated, and infected | *β_0_+ β_0_+β_1_X_i,1_+β_2_X_i,2_+β_4_+β_5.1_+β_8.1_* |
| Hispanic, not vaccinated, and not infected | *β_0_+β_1_X_i,1_+β_2_X_i,2_+β_5.1_* |
|  |  |
| Black, vaccinated, and infected | *β_0_+ β_0_+β_1_X_i,1_+β_2_X_i,2_+β_3_+β_4_+β_5.2_+β_6_+β_7.2_+β_8.2_+β_9.2_* |
| Black, vaccinated, and not infected | *β_0_+ β_1_X_i,1_+β_2_X_i,2_+β_3_+β_5.2_+β_7.2_* |
| Black, not vaccinated, and infected | *β_0_+β_1_X_i,1_+β_2_X_i,2_+β_4_+β_5.2_+β_8.2_* |
| Black, not vaccinated, and not infected | *β_0_+β_1_X_i,1_+β_2_X_i,2_+β_5.2_* |
|  |  |
| Other, vaccinated, and infected | *β_0_+β_1_X_i,1_+β_2_X_i,2_+β_3_+β_4_+β_5.3_+β_6_+β_7.3_+β_8.3_+β_9.3_* |
| Other, vaccinated, and not infected | *β_0_+ β_1_X_i,1_+β_2_X_i,2_+β_3_+β_5.3_+β_7.3_* |
| Other, not vaccinated, and infected | *β_0_+β_1_X_i,1_+β_2_X_i,2_+β_4_+β_5.3_+β_8.3_* |
| Other, not vaccinated, and not infected | *β_0_+β_1_X_i,1_+β_2_X_i,2_+β_5.3_* |

The other models for income, education, health status, and elderly status can be similarly derived, and the predicted probabilities can be similarly calculated. To facilitate interpretations, graphs will be used in presenting results in this appendix.

**Alternative Interpretations**

In this appendix, I use figures to depict changes in the probabilities of vaccination in the second year relative to the reference group: people not vaccinated and not infected by influenza in the first year. The corresponding values and standard errors are listed on the right side. It is an alternative way to interpret the coefficients in Table 3 in the manuscript.


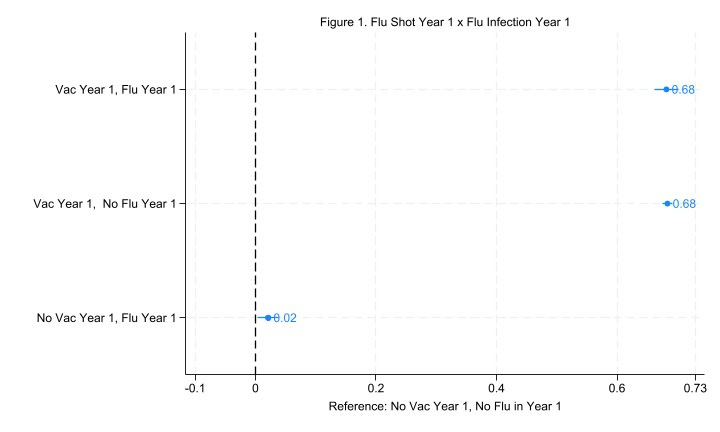


|  | Prob Change |
| --- | --- |
| Vac and Flu | 0.682*** |
|  | (0.010) |
| Vac and No Flu | 0.684*** |
|  | (0.004) |
| No Vac and Flu | 0.021** |
|  | (0.009) |

As seen in Figure 1 and the corresponding table, relative to people not vaccinated and not infected by influenza in the first year, individuals not vaccinated and infected in the first year have a 2% higher probability of vaccination in the second year. But the comparison between people vaccinated in the first year, i.e., the first two lines in the graph, shows that first-year vaccination has a much larger effect than the first-year flu infection.

Figure 2 is based on column three in Table 3 in the manuscript, i.e., results based on equation 2 with three-way interaction terms. Figure 2 shows the second-year vaccination probability changes relative to the reference group (white, not vaccinated, and not infected) by race and ethnicity.


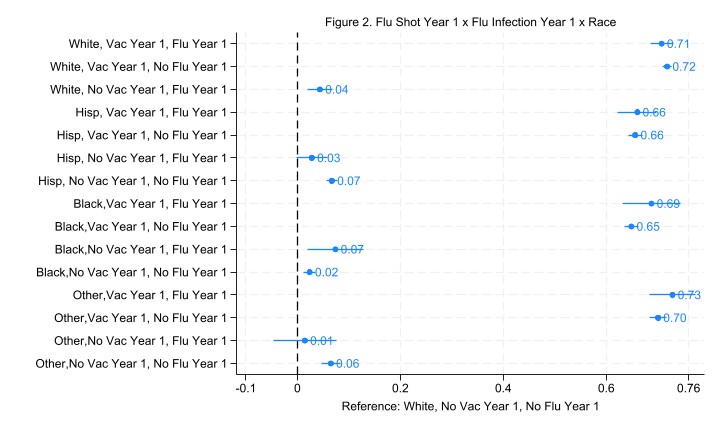


|  | Prob Change |  | Prob Change |
| --- | --- | --- | --- |
| White, Vac and Flu | 0.708*** | Black, Vac and Flu | 0.688*** |
|  | (0.012) |  | (0.029) |
| White, Vac, No Flu | 0.718*** | Black, Vac, No Flu | 0.649*** |
|  | (0.004) |  | (0.007) |
| White, No Vac, Flu | 0.044*** | Black, No Vac, Flu | 0.074*** |
|  | (0.012) |  | (0.027) |
| White, No Vac, No Flu | Reference | Black, No Vac, No Flu | 0.024*** |
|  |  |  | (0.006) |
| Hispanic, Vac and Flu | 0.660*** | Other, Vac and Flu | 0.729*** |
|  | (0.019) |  | (0.023) |
| Hispanic, Vac, No Flu | 0.656*** | Other, Vac, No Flu | 0.700*** |
|  | (0.007) |  | (0.008) |
| Hispanic, No Vac, Flu | 0.028* | Other, No Vac, Flu | 0.015 |
|  | (0.015) |  | (0.031) |
| Hispanic, No Vac, No Flu | 0.067*** | Other, No Vac, No Flu | 0.065*** |
|  | (0.006) |  | (0.010) |

We can compare the relative probability changes to examine the impact of previous vaccination and flu infection. Among vaccinated whites, flu infection is associated with a 1% (i.e., 0.718-0.708 = 0.01 = 1%) increase in the probability of vaccination in the second year compared to the reference group; among unvaccinated whites, flu infection is associated with a 4.4% increase in probability.

Among vaccinated black, the increase is 3.9% (0.688-0.649); among unvaccinated blacks, the increase is 5% (0.074-0.024). Among Hispanics, the increase is 0.4% (0.660-0.656) for the vaccinated, but for the unvaccinated, there is a 3.9% (0.028-0.067) decrease in the probability. Among vaccinated other races, the previous infection is associated with a 2.9% (0.729-0.700) increase in the probability of second-year vaccination; among unvaccinated other races, the increase is 5% (0.065-0.015).

Similarly, to derive the impact of first-year vaccination by race and ethnicity, we can compare the relative probability changes while holding the infection status fixed. Among flu-infected whites, those with first-year vaccination have a 66.4% (0.708-0.044) higher probability of second-year vaccination than those without first-year vaccination. Among whites who are not infected, those with first-year vaccination have a 71.8% higher probability than those without first-year vaccination.

Among flu-infected blacks, first-year vaccination is associated with an increase of 61.4% (0.688-0.074). Among blacks who are not infected, first-year vaccination is associated with an increase of 62.5% (0.649-0.024). Among flu-infected Hispanics, first-year vaccination is associated with an increase of 63.2% (0.660-0.028). Among Hispanics who are not infected, first-year vaccination is associated with an increase of 58.9% (0.656-0.067). Among non-Hispanic other races, the two increases are 71.4% (0.729-0.015) and 63.5% (0.700-0.065), respectively.

Figure 3 shows the second-year vaccination probability changes relative to the reference group (low income, not vaccinated, and not infected) by income categories. The corresponding values and their standard errors are listed below the figure.


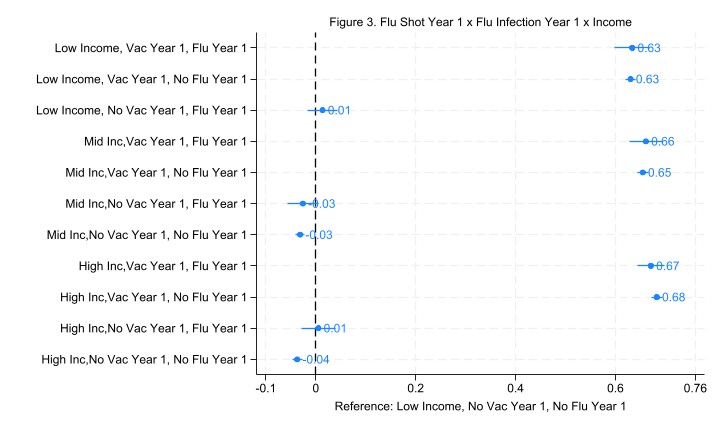


| Low Income | Prob Chng | Middle Income | Prob Chng | High Income | Prob Chng |
| --- | --- | --- | --- | --- | --- |
| Vac and Flu | 0.634*** | Vac and Flu | 0.661*** | Vac and Flu | 0.671*** |
|  | (0.018) |  | (0.017) |  | (0.014) |
| Vac, No Flu | 0.630*** | Vac, No Flu | 0.655*** | Vac, No Flu | 0.683*** |
|  | (0.005) |  | (0.006) |  | (0.006) |
| No Vac, Flu | 0.014 | No Vac, Flu | -0.026 | No Vac, Flu | 0.005 |
|  | (0.016) |  | (0.016) |  | (0.017) |
| No Vac, No Flu | Referece | No Vac, No Flu | -0.031*** | No Vac, No Flu | -0.037*** |
|  |  |  | (0.005) |  | (0.005) |

Among the low-income and vaccinated population, flu infection is associated with a 0.4% (0.634-0.630) increase in the second-year vaccination probability. Among the low-income and unvaccinated population, flu infection is associated with a 1.4% increase in the second-year vaccination probability. Among the middle-income population, the two increases are 0.6% (0.661-0.655) and 0.5% (-0.026-(-0.031)) for the vaccinated and the unvaccinated people, respectively. Among the high-income population, the two changes are -1.2% (0.671-0.683) and 4.2% (0.005-(-0.037)) for the vaccinated and the unvaccinated groups, respectively.

Among the low-income population that is infected with influenza, first-year vaccination is associated with an increase of 62% (0.634-0.014) in the second-year vaccination probability. Among the low-income population that is not infected with influenza, the increase is 63%. Among middle-income population, the increases are 68.7% (0.661-(-0.026)) and 68.6% (0.655-(-0.031)) for the infected and uninfected sub-population, respectively. Among the high-income population, the increases are 66.6% (0.671-0.005) and 72% (0.683-(-0.037)) for the infected and uninfected sub-population, respectively.

Figure 4 and the table below list the changes in second-year vaccination probability relative to individuals with less than a college education who are not vaccinated and not infected in the first year.

Among the vaccinated population with less than a college education, flu infection is associated with a decrease of 0.5% (0.667-0.672) in the probability of second-year vaccination. Among the unvaccinated population with less than a college education, flu infection is associated with an increase of 1.9% in the probability of second-year vaccination. Among people with college or higher levels of education, flu infection is associated with a 0.3% (0.727-0.724) increase for the vaccinated sub-group and a 2.8% (0.042-0.014) increase for the unvaccinated sub-group.


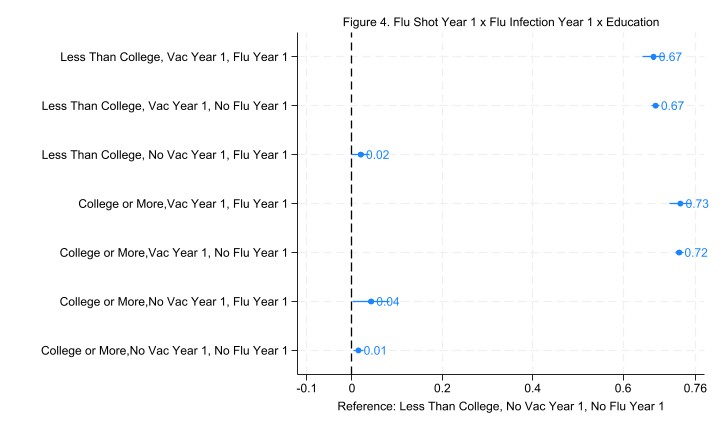


| Less than College | Prob Change | College or More | Prob Change |
| --- | --- | --- | --- |
| Vac and Flu | 0.667*** | Vac and Flu | 0.727*** |
|  | (0.012) |  | (0.012) |
| Vac, No Flu | 0.672*** | Vac, No Flu | 0.724*** |
|  | (0.004) |  | (0.005) |
| No Vac, Flu | 0.019* | No Vac, Flu | 0.042** |
|  | (0.010) |  | (0.021) |
| No Vac, No Flu | Reference | No Vac, No Flu | 0.014*** |
|  |  |  | (0.005) |

Figure 5 and the accompanying table list the changes in second-year vaccination probability by health status relative to individuals with poor or fair health that are not vaccinated and not infected in the first year.

Among vaccinated people who report poor or fair health, those infected with influenza have a 1.5% (0.648-0.663) lower probability of getting vaccinated in the following year than those not infected by influenza. Among the same health category, unvaccinated people infected with influenza have a 4.4% lower probability of getting vaccinated in the second year compared to unvaccinated people without the infection.

Among people with good health, influenza infection is associated with a decrease of 0.1% (0.641-0.642) for people vaccinated in the first year and an increase of 3.6% (-0.005-(-0.041)) for people not vaccinated in the first year. Among people with very good and excellent health, flu infection is associated with a 0.2% increase in probability for the vaccinated population and a 2.3% increase for the unvaccinated population.


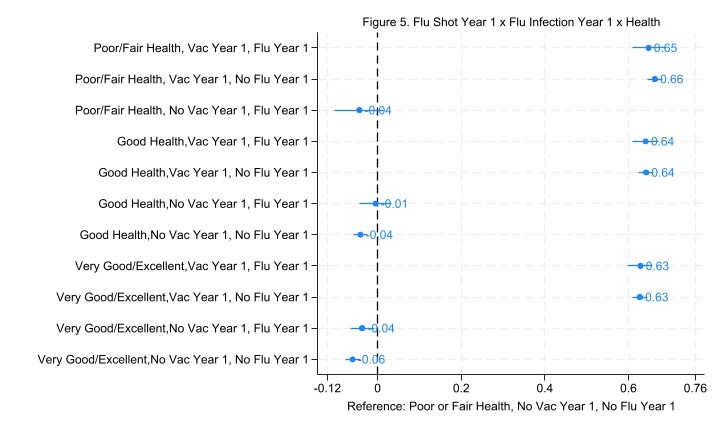


| Poor or Fair Health | Prob Chg | Good Health | Prob Chg | V. Good or Excel. | Prob Chg |
| --- | --- | --- | --- | --- | --- |
| Vac and Flu | 0.648*** | Vac and Flu | 0.641*** | Vac and Flu | 0.629*** |
|  | (0.020) |  | (0.016) |  | (0.015) |
| Vac, No Flu | 0.663*** | Vac, No Flu | 0.642*** | Vac, No Flu | 0.627*** |
|  | (0.009) |  | (0.010) |  | (0.010) |
| No Vac, Flu | -0.044 | No Vac, Flu | -0.005 | No Vac, Flu | -0.037** |
|  | (0.030) |  | (0.020) |  | (0.014) |
| No Vac, No Flu | Reference | No Vac, No Flu | -0.041*** | No Vac, No Flu | -0.060*** |
|  |  |  | (0.009) |  | (0.009) |

The last figure, Figure 6, shows the impact of first-year vaccination and flu infection on the second-year vaccination status by the elderly status. The probability changes by age group, first-year vaccination outcome, and infection history can be derived similarly to the previous analyses.


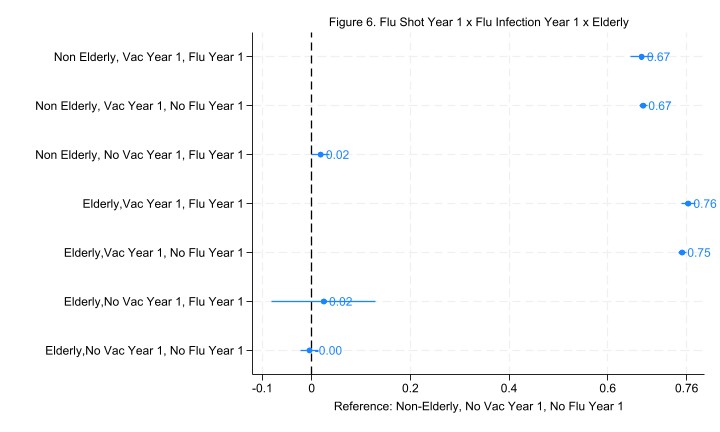


| Non-Elderly | Prob Change | Elderly | Prob Change |
| --- | --- | --- | --- |
| Vac and Flu | 0.669*** | Vac and Flu | 0.763*** |
|  | (0.012) |  | (0.007) |
| Vac, No Flu | 0.672*** | Vac, No Flu | 0.751*** |
|  | (0.004) |  | (0.004) |
| No Vac, Flu | 0.018* | No Vac, Flu | 0.025 |
|  | (0.009) |  | (0.054) |
| No Vac, No Flu | Reference | No Vac, No Flu | -0.004 |
|  |  |  | (0.009) |

**Robustness Tests**

To test the robustness of the results, I used logit models to re-estimate equations 1 and 2. Reported here are the estimated coefficients and predicted probabilities.

*Table 1A: Coefficients and Standard Errors from Logit Probability Models*

|  | (1) | (2) | (3) | (4) | (5) | (6) |
| --- | --- | --- | --- | --- | --- | --- |
| Vaccinated | 3.526*** | 3.847*** | 3.112*** | 3.419*** | 3.490*** | 3.369*** |
|  | (0.029) | (0.039) | (0.037) | (0.032) | (0.072) | (0.031) |
| Infected | 0.176*** | 0.353*** | 0.124 | 0.174** | -0.269 | 0.139** |
|  | (0.066) | (0.086) | (0.118) | (0.080) | (0.227) | (0.069) |
| Vaccinated # Infected | -0.197* | -0.481*** | -0.098 | -0.221* | 0.092 | -0.166 |
|  | (0.117) | (0.152) | (0.187) | (0.133) | (0.309) | (0.122) |
| Hispanic | 0.092** | 0.536*** | 0.099*** | 0.093** | 0.092** | 0.100*** |
|  | (0.038) | (0.046) | (0.037) | (0.037) | (0.038) | (0.037) |
| Non-Hispanic Black | -0.147*** | 0.190*** | -0.141*** | -0.144*** | -0.148*** | -0.133*** |
|  | (0.034) | (0.047) | (0.033) | (0.033) | (0.034) | (0.034) |
| Non-Hispanic Other Race | 0.192*** | 0.493*** | 0.201*** | 0.200*** | 0.192*** | 0.199*** |
|  | (0.054) | (0.064) | (0.054) | (0.055) | (0.054) | (0.053) |
| Middle Income | -0.064** | -0.067** | -0.240*** | -0.063* | -0.063* | -0.065** |
|  | (0.032) | (0.032) | (0.041) | (0.032) | (0.033) | (0.033) |
| High Income | 0.050 | 0.041 | -0.270*** | 0.048 | 0.051 | 0.050 |
|  | (0.035) | (0.035) | (0.042) | (0.035) | (0.035) | (0.036) |
| College or More | 0.297*** | 0.290*** | 0.292*** | 0.137*** | 0.296*** | 0.301*** |
|  | (0.030) | (0.031) | (0.031) | (0.041) | (0.030) | (0.030) |
| Good Health | -0.234*** | -0.234*** | -0.235*** | -0.234*** | -0.272*** | -0.245*** |
|  | (0.043) | (0.043) | (0.042) | (0.043) | (0.059) | (0.044) |
| Very Good or Excellent Health | -0.387*** | -0.389*** | -0.388*** | -0.388*** | -0.423*** | -0.394*** |
|  | (0.048) | (0.048) | (0.047) | (0.047) | (0.062) | (0.049) |
| Age 65 or Above | 0.535*** | 0.518*** | 0.539*** | 0.538*** | 0.535*** | -0.038 |
|  | (0.041) | (0.042) | (0.041) | (0.041) | (0.041) | (0.062) |
| Vaccinated # Hispanic |  | -1.047*** |  |  |  |  |
|  |  | (0.061) |  |  |  |  |
| Vaccinated # Non-Hispanic Black |  | -0.792*** |  |  |  |  |
|  |  | (0.072) |  |  |  |  |
| Vaccinated # Non-Hispanic Other |  | -0.701*** |  |  |  |  |
|  |  | (0.091) |  |  |  |  |
| Infected # Hispanic |  | -0.658*** |  |  |  |  |
|  |  | (0.157) |  |  |  |  |
| Infected # Non-Hispanic Black |  | 0.004 |  |  |  |  |
|  |  | (0.201) |  |  |  |  |
| Infected # Non-Hispanic Other |  | -0.717*** |  |  |  |  |
|  |  | (0.276) |  |  |  |  |
| Vaccinated # Infected # Hispanic |  | 0.799*** |  |  |  |  |
|  |  | (0.230) |  |  |  |  |
| Vaccinated # Infected |  | 0.409 |  |  |  |  |
| # Non-Hispanic Black |  | (0.349) |  |  |  |  |
| Vaccinated # Infected |  | 1.159*** |  |  |  |  |
| # Non-Hispanic Other |  | (0.405) |  |  |  |  |
| Vaccinated # Middle Income |  |  | 0.423*** |  |  |  |
|  |  |  | (0.062) |  |  |  |
| Vaccinated # High Income |  |  | 0.752*** |  |  |  |
|  |  |  | (0.067) |  |  |  |
| Infected # Middle Income |  |  | -0.062 |  |  |  |
|  |  |  | (0.181) |  |  |  |
| Infected # High Income |  |  | 0.188 |  |  |  |
|  |  |  | (0.165) |  |  |  |
| Vaccinated # Infected |  |  | 0.083 |  |  |  |
| # Middle Income |  |  | (0.283) |  |  |  |
| Vaccinated # Infected |  |  | -0.350 |  |  |  |
| # High Income |  |  | (0.278) |  |  |  |
| Vaccinated # College or More |  |  |  | 0.375*** |  |  |
|  |  |  |  | (0.058) |  |  |
| Infected # College or More |  |  |  | 0.025 |  |  |
|  |  |  |  | (0.159) |  |  |
| Vaccinated # Infected |  |  |  | 0.056 |  |  |
| # College or More |  |  |  | (0.254) |  |  |
| Vaccinated # Good Health |  |  |  |  | 0.041 |  |
|  |  |  |  |  | (0.074) |  |
| Vaccinated # Very Good or |  |  |  |  | 0.039 |  |
| Excellent Health |  |  |  |  | (0.074) |  |
| Infected # Good Health |  |  |  |  | 0.528** |  |
|  |  |  |  |  | (0.261) |  |
| Infected # Very Good or |  |  |  |  | 0.467* |  |
| Excellent Health |  |  |  |  | (0.242) |  |
| Vaccinated # Infected |  |  |  |  | -0.371 |  |
| # Good Health |  |  |  |  | (0.358) |  |
| Vaccinated # Infected |  |  |  |  | -0.281 |  |
| # Very Good or Excellent Health |  |  |  |  | (0.330) |  |
| Vaccinated # Age 65 or Above |  |  |  |  |  | 1.208*** |
|  |  |  |  |  |  | (0.082) |
| Infected # Age 65 or Above |  |  |  |  |  | 0.037 |
|  |  |  |  |  |  | (0.334) |
| Vaccinated # Infected |  |  |  |  |  | 0.715 |
| # Age 65 or Above |  |  |  |  |  | (0.451) |
|  |  |  |  |  |  |  |
| Observations | 103613 | 103613 | 103613 | 103613 | 103613 | 103613 |
| Standard errors in parentheses. * p<0.10, ** p<0.05, *** p<0.01; All models control for demographic, socioeconomic, health-related variables. Some are not reported here. Full results are available upon request. | | | | | | |

*Table 2A: Predicted Second-Year Vaccination Probability by Demographic, Socioeconomic, and Health*

| Pop. Group | Vac & Infection Status |  | Pop. Group |  |
| --- | --- | --- | --- | --- |
| Overall | Not Vac, Not Infected | 0.225 [0.215 0.235] | Less Than College | 0.209 [0.199 0.218] |
|  | Not Vac, Infected | 0.257 [0.231 0.282] |  | 0.238 [0.210 0.266] |
|  | Vac, Not Infected | 0.899 [0.893 0.904] |  | 0.879 [0.872 0.885] |
|  | Vac and Infected | 0.897 [0.880 0.913] |  | 0.874 [0.852 0.895] |
|  |  |  |  |  |
| White | Not Vac, Not Infected | 0.196 [0.185 0.206] | College or More | 0.232 [0.217 0.247] |
|  | Not Vac, Infected | 0.256 [0.223 0.289] |  | 0.268 [0.217 0.319] |
|  | Vac, Not Infected | 0.910 [0.904 0.916] |  | 0.923 [0.916 0.929] |
|  | Vac and Infected | 0.899 [0.879 0.920] |  | 0.925 [0.904 0.947] |
|  |  |  |  |  |
| Hispanic | Not Vac, Not Infected | 0.291 [0.276 0.306] | Poor or Fair Health | 0.269 [0.247 0.291] |
|  | Not Vac, Infected | 0.234 [0.188 0.279] |  | 0.220 [0.148 0.292] |
|  | Vac, Not Infected | 0.860 [0.849 0.871] |  | 0.915 [0.906 0.925] |
|  | Vac and Infected | 0.862 [0.831 0.893] |  | 0.901 [0.866 0.936] |
|  |  |  |  |  |
| Black | Not Vac, Not Infected | 0.227 [0.211 0.242] | Good Health | 0.220 [0.208 0.231] |
|  | Not Vac, Infected | 0.293 [0.225 0.361] |  | 0.266 [0.219 0.313] |
|  | Vac, Not Infected | 0.849 [0.838 0.860] |  | 0.896 [0.889 0.903] |
|  | Vac and Infected | 0.881 [0.833 0.930] |  | 0.894 [0.868 0.920] |
|  |  |  |  |  |
| Other | Not Vac, Not Infected | 0.282 [0.256 0.308] | Very Good or Excellent | 0.196 [0.185 0.206] |
|  | Not Vac, Infected | 0.216 [0.133 0.300] |  | 0.228 [0.198 0.258] |
|  | Vac, Not Infected | 0.892 [0.880 0.904] |  | 0.881 [0.875 0.888] |
|  | Vac and Infected | 0.918 [0.876 0.960] |  | 0.882 [0.858 0.906] |
|  |  |  |  |  |
| Low Income | Not Vac, Not Infected | 0.258 [0.245 0.271] | Less Than Age 65 | 0.194 [0.187 0.201] |
|  | Not Vac, Infected | 0.282 [0.236 0.328] |  | 0.216 [0.193 0.238] |
|  | Vac, Not Infected | 0.876 [0.868 0.884] |  | 0.862 [0.856 0.869] |
|  | Vac and Infected | 0.879 [0.850 0.907] |  | 0.859 [0.837 0.881] |
|  |  |  |  |  |
| Middle Income | Not Vac, Not Infected | 0.216 [0.20 20.229] | Age 65 or Above | 0.188 [0.169 0.206] |
|  | Not Vac, Infected | 0.226 [0.183 0.269] |  | 0.216 [0.113 0.318] |
|  | Vac, Not Infected | 0.894 [0.886 0.902] |  | 0.952 [0.946 0.958] |
|  | Vac and Infected | 0.898 [0.871 0.926] |  | 0.976 [0.962 0.991] |
|  |  |  |  |  |
| High Income | Not Vac, Not Infected | 0.211 [0.199 0.223] |  |  |
|  | Not Vac, Infected | 0.266 [0.224 0.308] |  |  |
|  | Vac, Not Infected | 0.919 [0.912 0.926] |  |  |
|  | Vac and Infected | 0.908 [0.884 0.933] |  |  |

The following figures are based on Table 2A. They present the predicted probabilities in Table 2A in graphs for easier comparison.


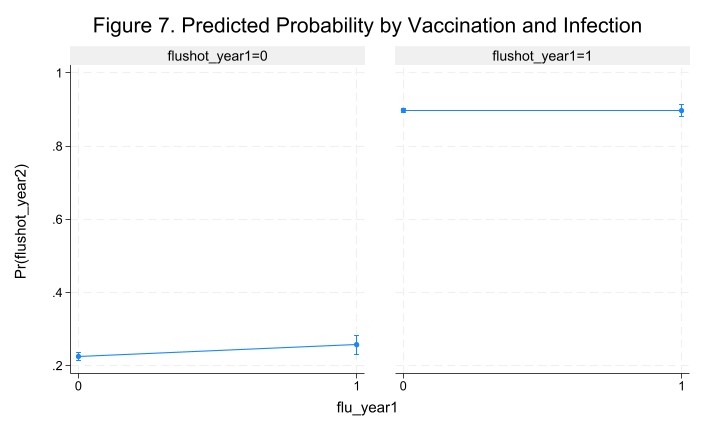


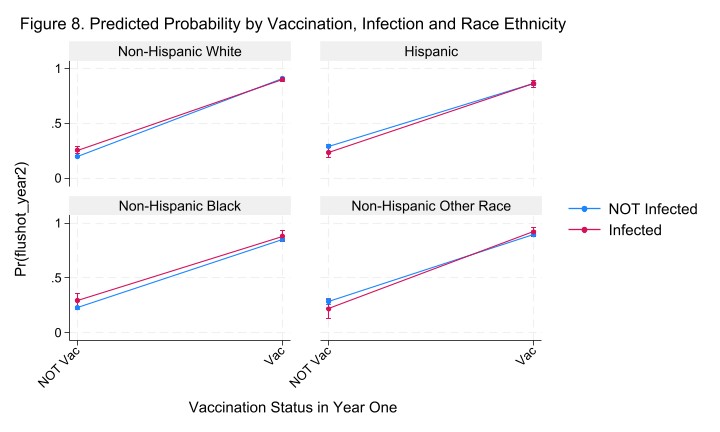


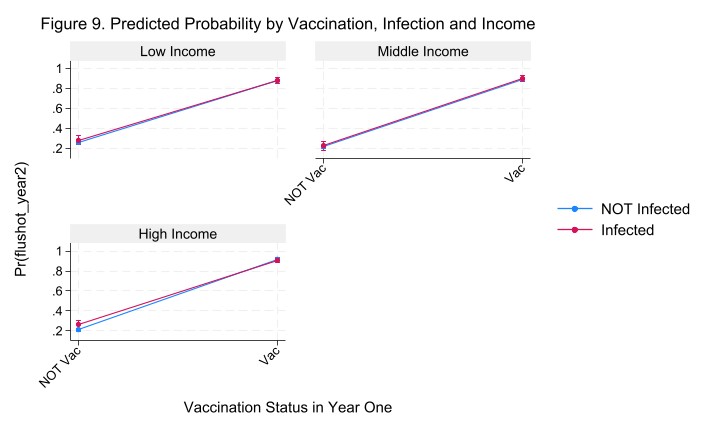


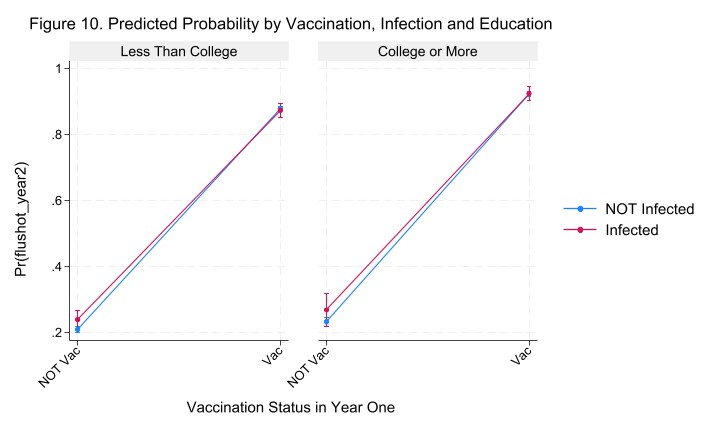


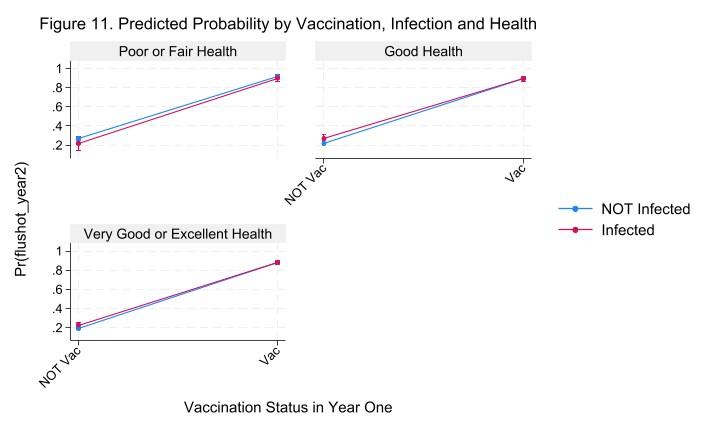


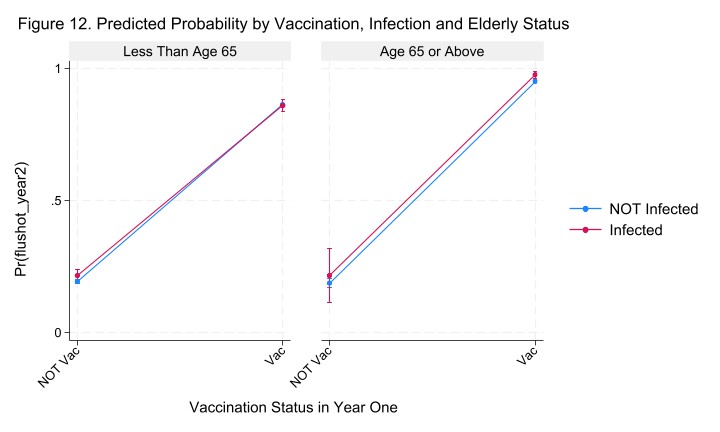


**Reference:**

1. Matjasko, J. L., Cawley, J. H., Baker-Goering, M. M., & Yokum, D. V. (2016). Applying behavioral economics to public health policy: illustrative examples and promising directions. *American journal of preventive medicine*, *50*(5), S13-S19.

2. Chen, F., & Stevens, R. (2017). Applying lessons from behavioral economics to increase flu vaccination rates. *Health promotion international*, *32*(6), 1067-1073.
